# IQ variability and cognitive life skills: insights from Spanish students in vocational physical activity and sports training

**DOI:** 10.3389/fspor.2025.1554023

**Published:** 2025-09-12

**Authors:** María Madrid-Rísquez, Virginia Alcaraz-Rodríguez, Pilar Gómez-Rey, Antonio Muñoz-Llerena

**Affiliations:** ^1^ Department of Education, Universidad Europea Miguel de Cervantes, Valladolid, Spain; ^2^ Research Group “Social Inclusion, Physical Education and Sport, and European Policies in Research” (HUM-1061), Universidad de Sevilla, Seville, Spain; ^3^ Faculty of Psychology and Education, Loyola University, Seville, Spain; ^4^ Department of Integrated Teaching, University of Huelva, Huelva, Spain; ^5^ Department of Didactics of Mathematics, Universidad de Sevilla, Seville, Spain; ^6^ Research Group “Technology & Human Interaction Interdisciplinary Hub (i-TECH)” (HUM-1138), Universidad de Sevilla, Seville, Spain; ^7^ Physical Education and Sports Department, Universidad de Sevilla, Seville, Spain

**Keywords:** vocational education, intelligence, IQ, WAIS-IV, sports

## Abstract

**Introduction:**

The increasing social and labor demand for technical professionals underscores the importance of vocational education in preparing students for the workforce. In the context of vocational education, Positive Youth Development can play a crucial role in enhancing students' cognitive and emotional life skills, which are essential for their overall development and future success.

**Methods:**

The objective of this study was to analyze the development of four cognitive areas (verbal comprehension, perceptual reasoning, working memory, and processing speed) alongside the Full Scale Intellectual Quotient (FSIQ) in the Intermediate Vocational Training Program in Outdoor Activity Guidance in Córdoba, Spain, and its relationship with cognitive areas using the WAIS-IV test. The sample included 176 students aged 16 -29, who were administered questionnaires under controlled conditions to ensure clarity and accuracy.

**Results:**

Results revealed that students generally scored below the population mean in overall IQ, with significant variability. Perceptual reasoning and working memory were average for most students, while verbal comprehension scores were predominantly low, and processing speed scores were very low. A moderate correlation was observed between FSIQ and verbal comprehension and perceptual reasoning, while processing speed showed a weak correlation.

**Discussion:**

These findings highlight the challenges faced by vocational students, particularly in areas critical for learning. The study concludes that while intelligence levels in this group are diverse, many students fall below average, emphasizing the need for tailored educational support. Future research should explore targeted interventions to enhance cognitive performance and success in vocational education pathways.

## Introduction

The increasing social and labor demand for technical professionals underscores the importance of vocational education in preparing students for the workforce. Vocational training programs, such as the Intermediate Vocational Training Program in Outdoor Activity Guidance, play a crucial role in equipping students with the necessary skills and knowledge to meet these demands. However, research in vocational education in Spain is alarmingly scarce, irregular, and underfunded, with limited institutional collaboration (1). This lack of research is further compounded by the declining reputation of Intermediate Vocational Training programs compared to Baccalaureate programs (2–4).

Positive youth development (PYD) emphasizes the importance of fostering the strengths and potential of young people through supportive environments and opportunities for growth (5, 6). In the context of vocational education, PYD can play a crucial role in enhancing students' cognitive and emotional skills, which are essential for their overall development and future success (7, 8). By integrating PYD principles into vocational training programs, educators can create a holistic learning experience that not only focuses on academic and technical skills but also promotes resilience, self-efficacy, and social competence. Furthermore, life skills acquisition is a fundamental aspect of PYD, encompassing a range of competencies such as critical thinking, problem-solving, communication, and teamwork (9). These skills are vital for students' personal and professional development, particularly in vocational settings where practical application of knowledge is key.

Research teams dedicated to vocational education are few and lack consolidation, with no effective communication network among them. Most studies in this field are descriptive, with small, non-random samples, and often rely on interviews and questionnaires that have not always been properly validated, raising concerns about the reliability of the findings (10).

The declining reputation of Intermediate Vocational Training programs has been influenced by legislative developments shaping formal education systems. Until recently, students pursuing the Baccalaureate viewed obtaining a university degree as a pathway to achieving higher social and economic status (11). Conversely, those choosing Intermediate Vocational Training programs often justified their decision by perceiving the Baccalaureate as more challenging (12). As a result, interest in Intermediate Vocational Training programs has generally been limited to students who faced greater difficulties during secondary education (13).

Professionals using educational or psychological assessments must ensure that these evaluations have a solid scientific foundation to support the conclusions and decisions derived from their administration (14). In clinical or educational contexts, where important decisions must be made to support children and adolescents, it is crucial to have rigorous information on the psychometric properties of widely used tests to ensure their appropriate, ethical, and fair use (15).

Incorporating life skills training into vocational education can help students navigate the complexities of the modern workforce, improve their employability, and contribute positively to their communities. Therefore, the aim of this study was to analyze the development of four cognitive areas [verbal comprehension (VCI), perceptual reasoning (PRI), working memory (WMI), and processing speed (PSI)] and a composite score representing Full Scale Intelligence Quotient (FSIQ), which is derived from standardized cognitive tests and provides an overall estimate of a person's general intellectual ability, to establish a cognitive profile of vocational training students and examine correlations among the areas. The main hypothesis of the study is that students enrolled in the Technical Degree in Physical and Sports Activities in Natural Environments exhibit high heterogeneity in the development of intelligence and different cognitive areas. In general, this group presents lower levels compared to the population mean.

## Materials and methods

### Research design

The study employed a cross-sectional quantitative design with descriptive and correlational objectives. Data collection involved individually administered tests to students enrolled in the Intermediate Vocational Training Program in Outdoor Activity Guidance.

### Sample

The study sample comprised 176 students from two public and three private vocational training centers in Andalusia, Spain. Participants ranged in age from 16 to 29 years (M = 17.5, SD = 1.7). A convenience, non-probabilistic sampling strategy has been utilized. Sample selection was intentional and driven primarily by accessibility criteria: each institution's administrative team facilitated access to facilities and coordination of schedules, and the geographic proximity enabled the researchers to travel efficiently and swiftly. In this way, data could be collected systematically under homogeneous conditions, while simultaneously ensuring compliance with the established ethical and methodological protocols. Inclusion criteria included enrollment in the Intermediate Vocational Training Program in Outdoor Activity Guidance and consent to participate (in the case of minors, parental consent was required). Exclusion criteria included lack of consent, diagnosed intellectual disability, severe neurodevelopmental disorders, or incomplete cognitive test protocols.

### Instrument

The WAIS-IV intelligence scale is a clinical test administered individually to evaluate the intelligence of individuals aged 16–89 years (16). It provides composite scores in specific cognitive areas such as VCI, PRI, WMI, and PSI. The dimensions of this test were the following:

(a) VCI: Assesses candidates’ ability to comprehend, express, and reason verbally, measuring their mastery of language.
(b) PRI: Examines the ability to process visual information, establish logical connections between ideas, and provide coherent responses based on such information.
(c) WMI: Analyzes cognitive capacity to comprehend, retain, and manipulate short-term information effectively.
(d) PSI: Measures the ability to quickly and efficiently process visual information and generate accurate responses.

The WAIS-IV demonstrates strong reliability coefficients, ranging from 0.72 to 0.93 for subtests and from 0.87 to 0.97 for indices and FSIQ (17). Testing was conducted individually, with each participant completing the mandatory 10 subtests. The duration ranged between 60 and 90 min.

Alongside with the WAIS-IV scale, a sociodemographic questionnaire was also administered, which included age, gender, and socioeconomic status, as these factors can influence cognitive performance and vocational development.

### Procedure

In order to carry out this study, the research team contacted the management of the educational center located at (Information to be included if the study is accepted), sending an information sheet with a brief description of the study, its objectives and the questionnaires to be used. Subsequently, once the approval of the school management was obtained, the first author went to the schools and administered the questionnaires to the students at the beginning of theory classes. The data collection took place in February 2020. Ethical approval and consent from the participants were obtained prior to data collection. Students were informed about the purpose and intended use of the instruments. Parental or legal guardian authorization was obtained for all participating minors. No adolescent was exposed to physical or psychological harm during testing. All data was kept confidential and not shared with third parties.

### Data analysis

Data were analyzed using SPSS software, version 20. For descriptive hypotheses, frequency distributions, central tendency measures, and variability measures were calculated. Normality in variable distributions was assessed, and in cases where assumptions were violated, alternative tests were conducted: Spearman's Rho for correlations and the Mann–Whitney *U*-test for sex-based differences. A cluster analysis was carried out to identify possible groups of students according to the IQ percentile scores. The cluster analyses were carried out using the guidelines proposed by Romesburg (18). The hierarchical cluster was analysed taking the Ward's Method, and for the similarity measures, the Euclidean distance squared was used. Then, a non-hierarchical cluster was done through the K-means method, taking as a reference the centroids of the cluster solutions of the hierarchical method for each period. Chi-square tests for qualitative variables (19), a MANOVA test for the continuous variables (20), and effect size (21) were calculated. The significance level was established at a value of *p* ≤ 0.05.

## Results

### Descriptive and correlation analysis

The WAIS-IV test evaluated the students’ IQ. Direct scores were transformed into percentiles based on population norms. As shown in Table 1, the average score (M = 37.39, SD = 23.3) indicates that the group, on average, scored below the population mean. Most students fell into the low or very low percentile ranges, with fewer cases in the higher ranges.

**Table 1:** Descriptive and correlational statistical analysis of quantitative variables from the WAIS-IV test.

Variables	M (*n* = 176)	SD	Skewness	Kurtosis	1. VCI	2. WMI	3. PRI	4. PSI
1. WAIS percentil VCI	41.04	32	0.352	−1.217				
2. WAIS percentil WMI	45.59	28	0.28	−1.309	-.314*			
3. WAIS percentil PRI	46.14	34	0.219	−1.451	0.127	0.007		
4. WAIS percentil PSI	28.63	24	0.864	−0.151	.252*	−0.15	.395*	
5. WAIS percentil FSIQ	37.39	23	0.218	−1.151	.611*	.234*	.632*	.594*

VCI, verbal comprehension; WMI, working memory; PRI, perceptual reasoning; PSI, processing speed; FSIQ, full scale intelligence quotient.

*Indicates that the result is statistically significant at the *p* < 0.01 level.

The distribution of scores across VCI, WMI, and PRI indicates that, on average, students scored below the population mean, with most scores in the mid or low ranges. For PSI, the group scored significantly below the population mean, with the majority in the very low range. Finally, a significant positive correlation was found between IQ and all cognitive domains (CVI, WMI, PRI, and PSI) at the 0.01 level (two-tailed). Table 2 provides a detailed breakdown of descriptive and correlational statistics.

**Table 2:** Cluster groups results.

Variable	High FSIQ (*n* = 24)	Moderate FSIQ (*n* = 53)	Low FSIQ (*n* = 43)	Very Low FSIQ (*n* = 56)	*F*	*p*-value	*η^2^*
	M (SD)	M (SD)	M (SD)	M (SD)			
WAIS percen VCI	76.16 (26.4)	50.64 (29.8)	40.63 (27.6)	17.21 (19.6)	32.950	<0.001*	0.365
WAIS percen PRI	63.75 (25.6)	62.57 (24.1)	43.19 (24.5)	23.59 (17.0)	33.581	<0.001**	0.369
WAIS percen WMI	51.72 (37.0)	57.05 (31.3)	42.19 (31.3)	36.48 (35.9)	3.817	0.011	0.062
WAIS percen PSI	54.13 (26.6)	37.21 (23.1)	23.28 (20.3)	13.70 (11.3)	27.829	<0.001***	0.327

VCI, verbal comprehension; WMI, working memory; PRI, perceptual reasoning; PSI, processing speed; FSIQ, full scale intelligence quotient.

* In the VCI there were statistically significant differences between all groups except between Group 1 and Group 2.

** Statistically significant differences between all groups except between Groups 2 and 3.

*** In the PSI there were statistically significant differences between all groups except between Group 1 and Group 4. η^2^, eta squared; small effect (.04), medium effect (.25), large effect (.64).

### Cluster and MANOVA analysis

The cluster analysis was determined based on the items of the percentile variable corresponding to the students IQ through a non-hierarchical analysis. The cluster analysis tested solutions for two, three, and four groups, with the four-group solution being the most suitable according to the results and objectives of this study. Cluster 1, labeled “*High FSIQ*”, comprised 13.6% of the sample and represented students with an IQ percentile of 74 (M = 6.92; SD = 0.9). Cluster 2, labeled “*Moderate FSIQ*”, comprised 30.11% of the sample and represented students with an IQ percentile near 54. Cluster 3, labeled “*Low FSIQ*”, comprised 24.43% of the sample and represented students with an IQ percentile near 30. Cluster 4, labeled “*Very Low FSIQ*”, comprised 31.81% of the sample and represented students with an IQ percentile near 11.

The MANOVA test showed that the independent variable, “*Intelligence (IQ percentiles)*”, significantly affected the combination of dependent variables (WMI, VCI, PSI, PRI) correlated with factors influencing intelligence (*Pillai's Trace: 0.954, F* *=* *15.83, p* ≤ *0.001, ηp^2^* *=* *0.318; Wilks' Lambda: 0.112, F* *=* *37.44; p* ≤ *0.001*). Assumption checks showed significant statistics for Box's M-test for homogeneity of covariance matrices (*χ^2^* *=* *102.191; p* ≤ *0.001*) and for the Shapiro–Wilk test for homogeneity (*SW* *=* *0.994; p* ≤ *0.001*).

The *F*-test showed moderate explanatory power of IQ for VCI (F = 32.950; *p* < 0.001), PRI (F = 33.581; *p* < 0.001), and PSI (F = 27.829; *p* < 0.001), while it was lower for WMI (F = 3.817; *p* = 0.011). A slight effect was observed across all variables, except for WMI, where the effect was minimal.

### Sociodemographic profile of the cluster

In relation to the analysis of the groups by cluster related to the variables of age and sex (Table 3), the highest percentage of women stands out in the highest percentiles (29.2%), in contrast to men where the highest percentage is found in very low percentiles. In relation to the variable “age”, statistically significant variables were found, where it stands out that the students with the lowest percentile are found in the youngest groups, with a slight effect.

**Table 3:** Sociodemographic cluster groups profile.

Variable	High FSIQ (*n* = 24)		Moderate FSIQ (*n* = 53)		Low FSIQ (*n* = 43)		Very low FSIQ (*n* = 56)	
	*N*	%	*N*	%	*N*	%	*N*	%
Sex [*χ*^2^ (3) = 2.68; *p* = 0.444; V = 0.123]								
Man	17	70.8	38	71.7	35	81.4	46	82.1
Woman	7	29.2	15	28.3	8	18.6	10	17.9
Age* [χ^2^ (4) = 19,11; *p* = 0.024; V = 0.190]								
16 years	9	37.5	16	30.2	13	30.2	7	12.5
17 years	9	37.5	21	39.6	9	20.9	15	26.
18 years	5	20.8	9	17.0	16	37.2	23	41.1
19 years or more	1	4.2	7	13.2	5	11.6	11	19.6

FSIQ, full scale intelligence quotient.

* *p* < 0.005; V: Cramer's V; Effect size: .07 (small), .21 (medium), .35 (large).

### Multiple regression analysis

A multiple linear regression was performed with the dependent variable IQ and the independent variables of the four cognitive dimensions analyzed in the WAIS-IV. The specific dimensions of the IQ explained 93.7% of the explained variance, according to the global percentile of the four cognitive dimensions, with a correlation of *r* = 0.968. The Durbin-Watson test showed a value of 2.23, being adequate to be less than three points, while the ANOVA test was statistically significant (F = 639.59; *p* < .001), which explains that there is a relationship between the different variables. The analysis of the regression assumptions was adequate for its performance.

Table 4 shows that the collinearity statistics were good, with values of tolerance between 0.1 and 1.0, while VIF were close to 1.0. The VCI was the dimension that has more influence in explain IQ (*β* *=* *0.59; t* *=* *28.57; p* *=* *0.001*), followed by PRI (*β* *=* *0.44; t* *=* *21.84; p* *=* *0.001*), WMI (*β* *=* *0.44; t* *=* *21.81; p* *=* *0.001*) and PSI (*β* *=* *0.34; t* *=* *16.30; p* *=* *0.01*).

**Table 4:** Multiple regression analysis.

Variable	*β*	*t*	*p*	Collinearity statistics	
				Tolerance	VIF
(Intercept)	−20.17	−15.80	<.001*		
WAISpercentilesVCI	0.59	28.57	<.001*	0.86	1.16
WAISpercentilesPRI	0.44	21.84	<.001*	0.88	1.13
WAISpercentilesWMI	0.44	21.81	<.001*	0.92	1.09
WAISpercentilesPSI	0.34	16.30	<.001*	0.83	1.21

FSIQ, full scale intelligence quotient. Dependent Variable FSIQ.

* *p* < .001.

## Discussion

The aim of this study was to analyze the development of four cognitive areas (VCI, PRI, WMI, and PSI) and a composite score representing FSIQ to establish a cognitive profile of vocational training students and examine correlations among the areas (22). The general hypothesis of this thesis stated that students who take the training cycle of Technician in Conducting Physical-Sports Activities in the Natural Environment exhibited considerable variability in the development of intelligence and the cognitive areas analyzed. In addition, it is postulated that, on average, these students present levels below the reference population average in both dimensions.

The following hypothesis was proposed: “*it is anticipated that, both in the cognitive areas and in intelligence, the students will exhibit high variability, with a small group in the medium-high level range and a larger group in the medium-low level range*”. The results confirmed the hypothesis presented, since there is high variability among the students studied. The distribution of subjects in the WAIS-IV intelligence test showed that 32.4% of subjects exceed the 50th percentile, and of these, 4.5% exceed the 75th percentile. In addition, 36.4% of subjects were below the 25th percentile.

These data indicated that the group, on average, is below the population average. There were more cases with low or very low scores than with high scores. This variability in the results must be due to the fact that a percentage of students who opt for Vocational Training have experienced academic failure at the secondary stage, which has limited the development of their intelligence for various reasons. In fact, in Spain, the school dropout rate was 31.75% in 2008, falling to 18.3% in 2017; In Europe, in 2008, the rate was 14.7%, falling to 10.6% in 2017.

Recent meta-analyses and neuroimaging studies support the use of cognitive interventions to improve working memory and processing speed (23–25). Low processing speed has been linked to poorer academic outcomes and challenges in adapting to workplace tasks requiring rapid responses. Interventions focusing on working memory and verbal comprehension are particularly important, as these areas strongly influence academic and job-related performance (26).

Based on these data, various studies on the causes of school failure and educational dropout emphasized that the economic factor was one of the most determining factors. However, the students in the sample came from a medium-high socioeconomic level, which suggests that there were other factors that influence the lack of motivation and interest of students, such as the type of educational institution, school organization, among others (27, 28). This situation highlighted the need to promote more technical and practical educational alternatives that prepare students for future job opportunities, an approach that could be offered by Vocational Training (29, 30), which may also include physical activity-based interventions that positively affect cognitive functions like working memory. Recent research reinforces this idea, pointing out that these alternatives were also the most socially fair to ensure that all people can participate in life with dignity (31–33).

The results obtained in the WMI, VCI, PSI and PRI confirmed the hypothesis presented since there was a high variability among students. Hartshorne and Germine (34) reported that working memory peaks around age 30 and then begins to decline, which underlines the importance of training and developing it to the maximum from childhood to adulthood. In addition, working memory had been identified as a good predictor of academic performance in the first years of compulsory schooling (34, 35). Allen et al. (36) and its importance persists into adolescence and adulthood, especially in language-related tasks (37–39).

Regarding the positive correlations found between intelligence and the different cognitive components (WMI, VCI, PSI, and PRI) assessed by the WAIS-IV, Dereili et al. (40) already demonstrated that cognitive training effectively affects recall and information processing speed in working memory and increases the ability to store verbal and visual information in working memory (41). Kim and Xin (42) had shown that learning to use strategies in learning is essential. Likewise, Fathi-Ashtiani et al. (43) indicated that teaching cognitive strategies improved information processing speed in students with learning disabilities. This topic was essential because cognitive skills are essential for correct information processing and students need to use these strategies to achieve academic success.

Lintunen et al. (44) analyzed the different subscales of the WAIS-IV, which independently predicted task success in different tasks performed in their study. For example, verbal comprehension predicted success on the information search and command line tasks, perceptual reasoning on the installation task, and working memory on the survey and spreadsheet tasks. Furthermore, the two variables assessing executive functions predicted success on online banking, postal service, and command line tasks. This suggests that cognitive domains contributed differently to performance, depending on the task. That is, they suggest that cognitive abilities, executive functions, and demographic factors had clear and independent contributions.

Also, Lintunen et al. (44) justified that the FSIQ is an aggregate measure, and the apparently rather weak dependence of the different tasks on other WAIS subscales, such as verbal comprehension and processing speed, meant that the combined score did not explain the data better. Finally, Ryan et al. (45) in their study demonstrated the relationship between the different WAIS-IV subscales and IQ (46).

## Limitations and prospective

All scientific endeavors bring valuable insights but also possess inherent limitations. In the present study, the socioeconomic homogeneity of the sample stands out as a primary constraint: all participants were drawn from an upper-middle-class background within a single region, limiting the generalizability of our findings to students from other income brackets and geographic areas. Moreover, the cross-sectional design, which captured cognitive performance at only one point in time, precludes any assessment of how those abilities evolve or respond to educational interventions over longer periods. A broader sample—both in size and scope, encompassing students from additional Spanish provinces—would not only strengthen statistical power but also allow for the investigation of regional differences. Although we employed the WAIS-IV to quantify intellectual functioning, focusing solely on IQ risks overlooking critical dimensions of students' development, such as motivational drivers, familial and social dynamics, and broader educational contexts. Lastly, the COVID-19 pandemic imposed logistical barriers that constrained our recruitment efforts and prevented the inclusion of a larger, more diverse cohort.

Looking ahead, several avenues promise to enrich and extend this line of research. Collaborative partnerships with vocational training instructors, interested universities, and industry sponsors could provide both practical expertise and funding to support expansive, multisite studies. Launching technology-enhanced entrepreneurial projects would further bridge theory and practice, applying research insights directly within vocational settings. The development of targeted educational interventions, particularly those designed to bolster verbal comprehension and processing speed, emerges as a clear priority, given our findings. To capture growth trajectories and intervention effects, future projects should adopt longitudinal designs that track cognitive and socioemotional development over months or years. Establishing interinstitutional research networks within the vocational training sector in Spain would address the current fragmentation in the field and foster sustained scholarly exchange. Finally, integrating frameworks such as PYD could offer a holistic lens through which to evaluate how structured, strength-based programming enhances both cognitive skills and broader life competencies among vocational training students.

## Conclusions

The conclusions linked to the established objectives are presented below, as well as those derived from the results obtained in relation to intelligence, cognitive areas and the interaction between both in the case of Vocational Training students of the Technical cycle in Conducting Physical-Sports Activities in the Natural Environment.

Vocational Training students of the Technical cycle in Conducting Physical-Sports Activities in the Natural Environment present, on average, a level of intelligence lower than the reference population average. A greater concentration of cases is observed in the categories of low or very low scores, compared to high scores. Likewise, a high heterogeneity is identified in the student body, evidenced by the coexistence of students with very low scores together with a small number of cases with high scores.

The level of the cognitive areas analyzed in the Vocational Training students presents a great variability. In the different areas, the cases are distributed at both extremes, with a few more cases in the very low than in the very high scores. In Verbal Comprehension Index, the majority of cases are concentrated in the low or very low score ranges when compared with the average performance of the reference population. This pattern suggests a significantly lower level of performance than expected compared to normative standards. Although some cases may be at medium or slightly higher levels, these represent a small proportion of the total.

About Working Memory Index, there is a notable variability in the level of development among students, with most cases concentrated in medium and medium-low score ranges, with a higher proportion of students in the low score categories compared to the high ones. In Perceptual Reasoning Index, on average, the level of development of this variable is below what is considered normative, with cases in the medium and medium-low ranges predominating.

The Processing Speed Index shows, on average, most cases are situated in the low or very low score ranges compared to the average performance of the reference population. Intelligence correlates significantly with the cognitive areas of Verbal Comprehension, Perceptual Reasoning, Working Memory and Processing Speed.

Finally, the regression analysis clarifies the fundamental role of developing the different cognitive areas for a possible improvement of the IQ. These findings validate the initial hypothesis, since the results show a high heterogeneity in the development of intelligence and different cognitive areas, which are related to each other, and they also explained 93.7% of the explained variance, according to the global percentile of the four cognitive dimensions.

## Data Availability

The raw data supporting the conclusions of this article will be made available by the authors, without undue reservation.
